# DOMINICA protocol: A study of benralizumab for severe eosinophilic asthma in children

**DOI:** 10.1111/pai.70272

**Published:** 2026-02-27

**Authors:** Theresa W. Guilbert, Maria Jison, Lena Börjesson Sjö, Viktoria Werkström, Hanna Grindebacke, Tomasz Durżyński, Aadarsh Lal, Jonathan Grigg

**Affiliations:** ^1^ Department of Pediatrics Cincinnati Children's Hospital and University of Cincinnati Cincinnati Ohio USA; ^2^ Late‐Stage Respiratory & Immunology, BioPharmaceuticals Research and Development AstraZeneca Gaithersburg Maryland USA; ^3^ Late‐Stage Respiratory & Immunology, BioPharmaceuticals Research and Development AstraZeneca Gothenburg Sweden; ^4^ Late‐Stage Respiratory & Immunology, BioPharmaceuticals Research and Development AstraZeneca Warsaw Poland; ^5^ Late‐Stage Respiratory & Immunology, BioPharmaceuticals Research and Development AstraZeneca Bengaluru Karnataka India; ^6^ Centre for Child Health, Blizard Institute Queen Mary University of London London UK

**Keywords:** benralizumab, biologics, eosinophils, exacerbation, pediatric patients, severe eosinophilic asthma, study design

To the Editor,

The treatment of severe asthma in pediatric patients presents significant challenges that need to be addressed. Asthma is defined as “severe” if it does not respond to a high dose of inhaled corticosteroid (ICS) plus long‐acting β_2_‐agonist (LABA) treatment, or if it worsens when the dose is reduced.^1^ Approximately 3% of children and adolescents with asthma in Europe have severe asthma.^2^

Patients with severe eosinophilic asthma (SEA) have increased levels of blood eosinophils and airway inflammation, which result in greater asthma exacerbation rates when compared to patients with a blood eosinophil count of <300 cells/μL.^3^ Benralizumab is a humanized afucosylated immunoglobulin G1 cytolytic monoclonal antibody that targets anti‐interleukin‐5 receptor α (anti‐IL‐5Rα), recruits natural killer cells and induces rapid and near complete depletion of eosinophils via antibody‐dependent cell‐mediated cytotoxicity.^4^ In two Phase 3 1‐year exacerbation studies (CALIMA and SIROCCO) and a 2‐year safety extension study (BORA), benralizumab significantly reduced exacerbation rates in patients with severe uncontrolled asthma when compared to placebo.^5^, ^6^, ^7^, ^8^ While improvements in asthma exacerbation rate were observed in adolescents on benralizumab in a pooled analysis of data from the CALIMA and SIROCCO studies, an improvement over placebo was not demonstrated due to a low crude exacerbation rate in the placebo group versus the benralizumab group.^9^ In the TATE study, the pharmacokinetics and long‐term safety of benralizumab in children with SEA were similar to outcomes observed in adolescents and adults.^10^

Benralizumab is approved as an add‐on maintenance treatment for SEA in pediatric patients aged ≥6 years in the United States, China, and Japan, and ≥12 years in Australia and New Zealand, and for severe asthma with an eosinophilic phenotype in adult patients in the European Union and other countries. Benralizumab is also approved for adults with eosinophilic granulomatosis with polyangiitis in several countries. Further investigation of benralizumab for SEA in pediatric patients is needed. Here, we present the protocol of the DOMINICA study, which will assess the efficacy and safety of benralizumab for SEA in pediatric and adolescent patients who experience frequent exacerbations despite the use of high‐dose ICS.

DOMINICA (NCT05692180) is a multicentre, randomized, double‐blind, placebo‐controlled, Phase 3 study that is currently recruiting patients. At least 200 patients are planned to be enrolled. See Table 1 for key eligibility criteria. Patients eligible to enroll in DOMINICA must: be aged 6 to <18 years; be diagnosed with SEA for at least 12 months prior to the first visit; have a diagnosis of severe asthma confirmed, evaluated and managed by the clinical site for at least 6 months prior to the first visit; be on a well‐documented, stable treatment for asthma with high‐dose ICS and at least one additional controller medication, such as LABA, leukotriene receptor antagonists, long‐acting muscarinic antagonists, or theophylline, for at least 6 months prior to the first visit; and have a history of asthma exacerbations of either (1) at least three exacerbations within the 12 months prior to the first visit, or (2) two exacerbations per year within 2 years prior to the first visit. Asthma exacerbations are defined as requiring systemic corticosteroid treatment and/or hospitalization. Key exclusion criteria include: presence of a clinically important pulmonary disease other than asthma, or past diagnosis of another pulmonary or systemic disease; life‐threatening asthma, defined as episodes requiring intubation associated with hypercapnia, respiratory arrest, hypoxic seizures, or asthma‐related syncopal episodes within 12 months prior to the first visit.

**TABLE 1:** Key inclusion and exclusion criteria for DOMINICA.

Key inclusion criteria	Aged 6 to <18 years at the time of signing the assent form and caregivers signing the informed consent form Physician diagnosis of severe eosinophilic asthma for at least 12 months prior to the first visit Diagnosis of severe asthma confirmed, evaluated, and managed by the clinical site for at least 6 months prior to the first visit History of asthma exacerbations defined as either: ≥3 exacerbations (requiring treatment with systemic corticosteroids and/or hospitalization) within 12 months prior to the first visit Two exacerbations per year within 2 years prior to the first visit and either stable maintenance oral corticosteroids for at least 3 months prior to the first visit OR at least one of the two exacerbations in the year prior to first visit resulting in hospitalization On well‐documented, stable treatment for asthma with high‐dose inhaled corticosteroids as specified in Global Initiative for Asthma (GINA) guideline/local guidelines/label requirements and at least one additional controller medication, such as long‐acting β_2_‐agonists, leukotriene receptor antagonists, long‐acting muscarinic antagonists, or theophylline for at least 6 months prior to the first visit
Key exclusion criteria	Clinically important pulmonary disease other than asthma (including but not limited to: active lung infection, chronic obstructive pulmonary disease, bronchiectasis, pulmonary fibrosis, cystic fibrosis, hypoventilation syndrome associated with obesity, lung cancer, alpha 1 anti‐trypsin deficiency, and primary ciliary dyskinesia) or patients who have a previous diagnosis of pulmonary or systemic disease, other than asthma, associated with elevated peripheral eosinophil counts (e.g., allergic bronchopulmonary aspergillosis/mycosis, eosinophilic granulomatosis with polyangiitis, and hyper‐eosinophilic syndrome) Life‐threatening asthma (defined as episodes requiring intubation associated with hypercapnia, respiratory arrest, hypoxic seizures, or asthma‐related syncopal episodes within 12 months prior to the first visit)

The design of the DOMINICA study is summarized in Figure 1. Throughout the study, all patients will receive background standard‐of‐care treatment. Patients will be randomized 1:1 during the double‐blind period (DBP) to receive benralizumab or placebo subcutaneously every 4 weeks (Q4W) for three doses, then every 8 weeks (Q8W). Patients in the benralizumab group will be given benralizumab 30 mg if they are aged ≥12 years, or aged <12 years with a weight of ≥35 kg; all other patients will receive benralizumab 10 mg. If a patient in the benralizumab group turns 12 years old or their weight becomes ≥35 kg during the study, the patient will receive benralizumab 30 mg at all subsequent treatment visits. The minimum duration of the DBP for each patient will be 16 weeks and will continue until a protocol‐defined asthma exacerbation is experienced or the pre‐defined number of exacerbations are observed in the DBP (≥111 patients with an asthma exacerbation event), whichever comes first. The “time to first asthma exacerbation event” design for the DBP will ensure patients will not continue to experience exacerbations for a prolonged period whilst on placebo. Patients who experience an asthma exacerbation will be eligible to join the open‐label extension (OLE), which will last at least 48 weeks for patients aged ≥12 years and at least 104 weeks for patients aged <12 years. Dosing of benralizumab during the OLE will be determined the same way as the DBP. Patients who received benralizumab during the DBP will receive one dose of benralizumab subcutaneously at the start of the OLE (Week 0), then one dose of placebo at Week 4, then benralizumab Q8W from Week 8. Patients who received placebo during the DBP will receive benralizumab subcutaneously Q4W for three doses then Q8W. At the end of treatment, patients will be seen for a follow‐up appointment 8 weeks after the last dose of investigational product.

**FIGURE 1:**
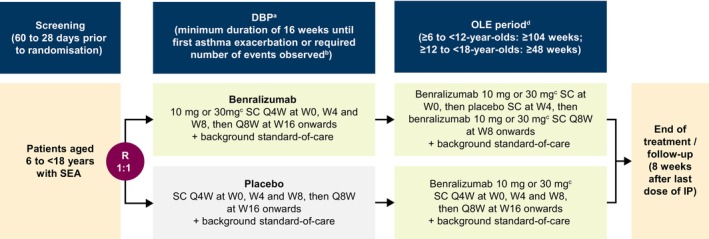
Design of the DOMINICA study. ^a^Randomization of patients will be stratified by age (6 to <12 years and ≥12 to <18 years). ^b^The duration of the DBP for each patient will be ≥16 weeks, and will continue until the patient experiences a protocol‐defined asthma exacerbation or the required number of exacerbations are observed in the DBP (≥111 patients with an asthma exacerbation event, including ≥30 patients aged between ≥6 and <12 years and ≥80 patients aged between ≥12 and <18 years). ^c^Benralizumab 30 mg will be given to patients aged ≥12 years and those aged <12 years with a weight of ≥35 kg; all other patients will receive benralizumab 10 mg. If a patient turns 12 years old or their weight becomes ≥35 kg during the study, the patient will receive benralizumab 30 mg at all subsequent treatment visits. ^d^The duration of the OLE period is based on the age of the patient at randomization. Abbreviations: DBP, double‐blind period; IP, investigational product; OLE, open‐label extension; Q4W, every 4 weeks; Q8W, every 8 weeks; R, randomization; SC, subcutaneous; SEA, severe eosinophilic asthma; W, week.

The primary endpoint of the DOMINICA study is time to first protocol‐defined asthma exacerbation, defined as worsening of asthma that requires a medical intervention. Worsening of asthma is defined as at least one of the following: worsening of asthma signs/symptoms; increased use of “as needed” reliever medication; deterioration of lung function (i.e., peak expiratory flow, forced expiratory volume in 1 s). Secondary endpoints will assess the: effect of benralizumab on asthma control and symptoms, health‐related quality of life and pulmonary function during the DBP; annualized asthma exacerbation rate (AAER) in the DBP; pharmacokinetics and immunogenicity of benralizumab. Exploratory endpoints will assess predictors of benralizumab response and the effect of benralizumab on blood biomarkers. Safety and tolerability of benralizumab will also be assessed. The OLE will further assess the AAER as well as the safety and tolerability of benralizumab. See Table 2 for a summary of all primary, secondary, exploratory, and safety endpoints.

**TABLE 2:** Endpoints for DOMINICA.

Primary endpoint	Time to first protocol‐defined asthma exacerbation, defined as worsening of asthma that requires a medical intervention. At least one of the following three elements must be fulfilled to meet the definition of asthma exacerbation: worsening of asthma signs/symptoms increased use of "as needed" reliever medication deterioration of lung function (i.e., PEF, FEV_1_) A medical intervention for asthma exacerbations is defined as follows: A temporary bolus/burst of systemic corticosteroids or a temporary increase in stable oral corticosteroid background dose for ≥3 consecutive days to treat symptoms of asthma worsening. A single depo‐injectable dose of corticosteroids will be considered equivalent to a 3‐day bolus/burst of systemic corticosteroids An emergency room visit (defined as evaluation and treatment for <24 h in an emergency department) due to asthma that required systemic corticosteroids (as per the above) An in‐patient hospitalization due to asthma (defined as admission to an inpatient facility and/or evaluation and treatment in a healthcare facility for ≥24 h)
Secondary endpoints	Change from baseline during the DB treatment period in the following measures: Interviewer‐administered version of the Asthma Control Questionnaire Asthma symptoms score (reported using the PASO questionnaire or Asthma Daily Diary) Rescue medication use (reported using the PASO questionnaire or Asthma Daily Diary) Night‐time awakenings (reported using the PASO questionnaire or Asthma Daily Diary) Peak expiratory flow Pediatric Asthma Quality of Life Questionnaire – Interviewer Administered Pre‐dose/pre‐bronchodilator FEV_1_ and post‐bronchodilator FEV_1_ at the site AAER in the DB treatment period Pharmacokinetics of benralizumab (serum trough concentration) Immunogenicity of benralizumab (anti‐benralizumab antibodies)
Exploratory endpoints	Baseline levels and change from baseline in: Eosinophil granule proteins Blood biomarkers (e.g., transcriptomics and protein biomarkers) Baseline levels of serum biomarkers of atopy (e.g., total and specific immunoglobulin E)
Safety endpoints	Safety assessments including AEs and serious AEs: Occurrence/frequency Relationship to benralizumab as assessed by the investigator Intensity Seriousness Death AEs leading to discontinuation of benralizumab Vital sign data Clinical laboratory parameters
OLE period endpoints	AEs and serious AEs AAER

*Note*: All endpoints are versus placebo.

Abbreviations: AAER, annualized asthma exacerbation rate; AE, adverse event; DB, double‐blind; FEV_1_, forced expiratory volume in 1 s; OLE, open‐label extension; PASO, Pediatric Asthma Symptom Observer‐reported; PEF, peak expiratory flow.

In conclusion, the Phase 3 DOMINICA study is designed to assess the efficacy and safety profile of benralizumab for SEA in pediatric patients and to provide further evidence to guide the treatment of SEA in patients aged <18 years. The study will ensure patients with SEA are not on placebo for a prolonged period, as the primary outcome of the study is “time to first asthma exacerbation event”. Data from the DOMINICA study may provide evidence for a new treatment option for pediatric patients with SEA, and will complement findings from CALIMA, SIROCCO, and TATE.^5^, ^6^, ^10^ At the time of writing, the DOMINICA study is recruiting and is estimated to complete in 2032.

## AUTHOR CONTRIBUTIONS

**Theresa W. Guilbert:** Investigation; writing – review and editing. **Maria Jison:** Conceptualization; methodology; investigation; writing – original draft; writing – review and editing; supervision; project administration; funding acquisition. **Lena Börjesson Sjö:** Writing – review and editing; supervision; writing – original draft. **Viktoria Werkström:** Conceptualization; methodology; investigation; writing – original draft; writing – review and editing; supervision; project administration. **Hanna Grindebacke:** Methodology; writing – review and editing; writing – original draft. **Tomasz Durżyński:** Conceptualization; methodology; writing – review and editing; supervision. **Aadarsh Lal:** Software; formal analysis; validation; writing – review and editing. **Jonathan Grigg:** Methodology; writing – review and editing.

## FUNDING INFORMATION

DOMINICA (NCT05692180) is funded by AstraZeneca.

## CONFLICT OF INTEREST STATEMENT

Theresa W. Guilbert declares employment at Cincinnati Children's Hospital Medical Center; grants/funds from Amgen, AstraZeneca, GlaxoSmithKline, National Institute of Health, OMPharma, Regeneron, Sanofi; advisory council or committee member for AstraZeneca, Genentech, OMPharma, Regeneron, Sanofi, TEVA; honoraria from Advent, Acme Pharma, PlatformQ Health, Sanofi; royalties from UpToDate; BPCA and DSMP member for the Best Pharmaceuticals for Children Act. Maria Jison, Lena Börjesson Sjö, Viktoria Werkström, Hanna Grindebacke, Tomasz Durżyński, and Aadarsh Lal declare employment at AstraZeneca and may hold stocks and/or stock options. Jonathan Grigg declares honoraria from AstraZeneca.
